# Post-Partum Complications

**Published:** 1890-06

**Authors:** A. E. Aust Lawrence

**Affiliations:** Physician-Accoucheur to the Bristol General Hospital


THE BRISTOL

Medico-Chirurgical Journal.

JUNE, 1890.

### POST-PARTUM COMPLICATIONS.

Read before the Bristol Medico-Chirurgical Society, April 9th, 1890,

BY

A. E. AUST LAWRENCE, M.D.,

*Physician-Accoucheur to the Bristol General Hospital.*

---

MR. PRESIDENT AND GENTLEMEN,—I wish to bring before you to-night some of the abnormal conditions which may arise *after the birth of the child and placenta.* I do not, of course, intend that this paper should be in any way exhaustive of the subject, but simply to lay before you certain post-partum abnormalities which, although not of frequent occurrence, yet occur sufficiently often to merit attention.

There is no doubt but that one complication will present itself to your minds at once; viz., *post-partum hæmorrhage.* Of this I may say that, as I consider the treatment of it should be mainly by *prevention,* I

shall do no more than refer you to a paper I read and published some time ago, upon the "Prevention of Post-partum Hæmorrhage;" and I will only allude under this heading to three valuable items in treatment I find so often overlooked:

1. The thorough clearing-out of the uterus by passing the hand into that organ whenever the amount of blood lost post-partum is abnormal.
2. The splendid results obtained in securing and maintaining uterine contraction by the intra-uterine injection of hot water at 110°, rendering the use of the iron solution scarcely ever necessary.
3. The subcutaneous injection of Bonjean's ergotin into the cellular tissue of the abdominal walls. This mode of injection I have practised for at least fifteen years, and have never seen ill results from it either locally or otherwise.

*Secondary post-partum hæmorrhage* may be a very serious complication when due to the detachment of a piece of placenta, or a secondary placenta which was not detached at the birth. These separate masses of placenta are known under the name of Placentæ succenturiatæ. I saw one case where, on the tenth day, most alarming hæmorrhage set in; and I removed a distinct lobule of placenta, which I have no doubt was a separate cotyledon, as the medical man assured me the placenta came away whole and entire. The reason of the profuse hæmorrhage was no doubt due to the uterus having shrunk down to a point incompatible with the proper surface for the portion of the placenta left, and hence partial detachment and profuse hæmorrhage.

The removal of the piece of placenta caused immediate arrest of the hæmorrhage.

*Concealed Post-partum Hæmorrhage.*—I have seen four cases where the child has been born and the placenta delivered, and the patient left at the end of one hour with the uterus well contracted, pulse below 100, and the discharge normal; and yet within three or four hours afterwards I have been called to see these patients apparently dying from hæmorrhage, pale and pulseless, yet no external evidence of hæmorrhage. On removing the binder, the distended abdomen at once gave the clue to the condition, and on passing the hand into the uterus I was enabled to clear out an enormous quantity of coagulated blood; prompt injections of hot water secured uterine contraction, and the women were saved.

I was told of a similar case the other night by Dr. Champneys, of London.

*Ruptured Perineum and Labium.*—I allude to these cases for two reasons: first, as I have seen three cases where most alarming hæmorrhage occurred from ruptured perineum and labium; and, secondly, to urge the routine practice of stitching up the torn perineum.

I will very briefly allude to one case where a deep laceration of the left labium nearly caused fatal hæmorrhage.

I attended the case myself, and congratulated myself on there being no laceration of the perineum. I left the woman one hour after the labour was over, and she then was all right. I saw her three hours afterwards, and found her very pale and restless. I was told there was no discharge of blood, and there was none on the diaper except a stain; but the woman lay on her left side, well over on her chest, and the diaper was fixed rather tightly from the perineum up to the pubes. The result of this was, that the blood was directed up on to the abdomen and filled the bed without appearing in the usual place.

The lesson this teaches is, always to insist upon women lying on their *backs*, and not to have any diaper brought up over the external genitals for at least twenty-four hours: let it be placed simply under them, to catch the discharge.

I believe some of the cases of concealed post-partum hæmorrhage I have mentioned were brought about by the women being allowed to lie on their sides. I believe if you put a small pad over the pubes and a very tight binder, and let the woman lie well over on her left side, you will go a long way towards inducing concealed post-partum hæmorrhage—just as you can produce retroflexion post-partum by keeping a woman on her back for three or four weeks with a hypogastric pad and a tight binder.

*Post-partum Nausea.*—I have seen six cases where the most alarming symptoms have been caused simply by *nausea*, and in all cases free and thorough emptying of the stomach caused all the alarming symptoms to disappear. Two of the women had had chloroform, and four had not.

All the confinements were natural. From about thirty minutes to one hour after delivery the symptoms set in, with a feeling of faintness, giddiness, loss of sight, and general restlessness; no hæmorrhage either external or intra-uterine. Stimulants were freely given both by mouth and hypodermically, but the symptoms persisted, and the state of the patient was most alarming until suddenly the stomach evacuated its contents—generally a very large amount,—then immediate relief was experienced, and the dangerous symptoms disappeared. These cases are so alarming that I have known very experienced obstetricians utterly at a loss to account for the symptoms, and not at all disposed to think that nausea could produce such a condition.

*Shock and Uterine Colic the result of Vaginal Injections.* —I have seen several cases where women after their confinements have been doing very well until one day when receiving a vaginal injection from the nurse they have suddenly been seized with acute abdominal pain, shock, and collapse, with several shivering fits. There has always been a distinct connection with the giving of the vaginal injection and the onset of the symptoms. In the majority of the cases all the symptoms have disappeared in twenty-four hours. I believe that the cause is the sudden distension of the uterus by a little fluid which has gained access to it. I do not think it is caused by fluid passing along the Fallopian tubes, as the symptoms disappear so rapidly, and also because I produced exactly the same train of symptoms by letting a Barnes' bag distend in the cavity of the body of the uterus, where it had slipped from the cervical canal. In this case nothing could have passed along the tubes.

I have given up the use of vaginal injections post-partum, and have substituted the use of iodoform pessaries.

I may mention that Dr. Graily Hewit has recently introduced glass tubes for vaginal and intra-uterine injections, especially prepared to allow a free return current by means of a deep groove on the sides of the tubes.

*Bladder Cases.*—Everyone knows that retention of urine is a post-partum complication in some women; but I do not allude to the cases which we meet with usually, but to some bladder complications which, although uncommon, may yet occur to any of our patients.

First case, due to the bad habit of keeping a woman *persistently* on her left side. I was sent for one evening to see Mrs. B., who had been confined four days. She had great abdominal pain, a freely-fluctuating tumour in the middle line reaching from the umbilicus to the pubes, and a large solid tumour reaching from the crest of the ilium up to her ribs on that side. I was told she passed her water "regularly."

The *regular* passing of water consisted in a more or less persistent dribble.

The *cystic tumour* disappeared on the passing of the catheter.

The *solid tumour* was the uterus, and when the woman was put on her back it assumed its proper position; and as she was allowed to remain on her back, and only had very slight pressure with her binder, there was no more trouble with the water.

The *second* case was rather puzzling; for although the medical attendant suspected bladder distension from the dribbling of urine, yet the evident enlargement of the abdomen was absolutely resonant in front. I found this was the case; but there was a freely-fluctuating abdominal tumour, dull at the sides, but resonant in front. A catheter removed two pints and a half of urine, and the cystic tumour disappeared. There was no doubt here—intestine fixed in front of the bladder.

The *third* case was an over-distended bladder, containing two pints of urine; and yet this woman passed half-a-pint of urine every six hours. I was asked to see her because she evidently had an increasing abdomen, with pain; and the bladder condition was not suspected, as the urine was passed apparently in a normal manner.

I simply mention these cases as they are a little out of the common.

